# An Immunophenotyping of Ovarian Cancer With Clinical and Immunological Significance

**DOI:** 10.3389/fimmu.2018.00757

**Published:** 2018-04-10

**Authors:** Kai Yang, Weiwei Zhao, Ge Lou, Zhiwei Rong, Huan Xu, Wenjie Wang, Wei Song, Yuqing Cai, Yan Hou, Kang Li

**Affiliations:** ^1^Department of Epidemiology and Biostatistics, School of Public Health, Harbin Medical University, Harbin, China; ^2^Department of Gynecology Oncology, The Tumor Hospital, Harbin Medical University, Harbin, China

**Keywords:** immunophenotyping, immune checkpoint blockade, ovarian cancer, gene expression, tumor microenvironment, *PD-1*, *CTLA-4*

## Abstract

Immune checkpoint blockade (ICB), mainly anti-CTLA-4 and anti-PD-1/PD-L1 therapy, has showed promising clinical benefits in the treatment of some cancer types; however, its application in ovarian cancer is still in the primary stage. Immunophenotyping can help us understand the clinical characteristics and immune status of cancer, and thus benefit immunotherapy and personalized therapy. In this study, we clustered 907 ovarian cancer patients into three immune molecular subtypes (IMMSs) based on 48 genes. Expression data were downloaded from the Gene Expression Omnibus database. Unsupervised consensus clustering was used to identify IMMS. Clinical and immunological characteristics and gene expression patterns of different IMMS were compared, and associations between IMMS and tumor microenvironment immune types were explored. Three IMMSs with different clinical and immunological characteristics were identified, in which type I and II ovarian cancer patients were similar to each other. There were more serous and low-grade tumors in type I and II ovarian cancer. IMMS was associated with disease-free survival before and after adjusting for clinical characteristics and ICB-related genes. Among the differentially expressed genes identified in our study, about 90% (25/28) were highly expressed in type I and II ovarian cancer. Genes related to ICB (*CTLA-4, PD-L1*, and *PD-L2*) and cytotoxic lymphocytes (*CD8A, GZMA*, and *PRF1*) were all highly expressed in type I and II ovarian cancer. Patients with type I and II ovarian cancer may be more sensitive to anti-CTLA-4 therapy, anti-PD-1/PD-L1 therapy, and a combination of immunotherapies. In contrast, patients with type III ovarian cancer may be insensitive to these treatments and require new therapies.

## Introduction

Immune checkpoint blockade (ICB) is a new strategy of immunotherapy that has shown promising clinical benefits in the treatment of some cancer types (1, 2). Anti-CTLA-4 and anti-PD-1/PD-L1 therapy are the two main cancer treatments for ICB. In 2011, the US Food and Drug Administration (FDA) approved the first checkpoint-blocking antibody Yervoy, an anti-CTLA-4 antibody, for the treatment of patients with advanced melanoma (3). In 2014, the FDA approved the first anti-PD-1 antibody, Keytruda, for advanced melanoma (4). After that, the FDA approved application of this drug to other types of cancers, such as advanced non-small cell lung cancer and head and neck squamous cell cancer (5, 6). After these breakthroughs, new checkpoint-blocking antibodies (e.g., Opdivo, Tecentriq, Imfinzi, and Bavencio) have been approved or are undergoing clinical trials for many other cancers (e.g., breast cancer, colorectal cancer, and gastric cancer) (7–10).

Ovarian cancer is the most common cause of gynecological cancer-associated deaths in developed countries (11, 12). Clinical trials of anti-CTLA-4 antibody (NCT02571725), anti-PD-1/PD-L1 antibody (NCT02498600 NCT02674061), or a combination of these (NCT03249142) are undergoing for ovarian cancer. Therefore, it is important to evaluate and understand the response to ICB for ovarian cancer patients. According to previous study, *CTLA-4, PD-L2, GZMA*, and *PRF1* genes were related to anti-CTLA-4 therapy, and *PD-L1, CD8A, GZMA*, and *PRF1* genes were related to anti-PD-1/PD-L1 therapy (13–15).

The sensitivity to anti-PD-L1 therapy can also be evaluated by tumor microenvironment immune types (TMIT), which is a tumor classification method based on the *PD-L1* status and the presence or absence of tumor-infiltrating lymphocytes (TILs) (16). TIL was represent by RNA-based metric of immune cell cytolytic activity (CYT) proposed by Rooney et al. (17). CYT can be measured by calculating the geometric mean of expression of two cytolytic effectors, *GZMA* and *PRF1*. Tumors can be divided into four TMITs based on expression levels of *PD-L1* and *CD8A*/CYT as follows: TMIT I, *PD-L1* high expression and *CD8A*/CYT high expression; TMIT II, PD-L1 low expression, and *CD8A*/CYT low expression; TMIT III, *PD-L1* high expression, and *CD8A*/CYT low expression; and TMIT IV, *PD-L1* low expression, and *CD8A*/CYT high expression. According to TMIT, various combination cancer therapy approaches can be considered. This stratification of cancers, which is applied to stratified cancer into four types based on their immune reactions, sets a framework to identify which pathways should be targeted to elicit the best response for each tumor type (18).

In our study, we analyzed 907 ovarian cancer patients from eight Gene Expression Omnibus (GEO; https://www.ncbi.nlm.nih.gov/geo/) datasets measured by the Affymetrix Human Genome U133A Plus 2.0 Array microarray platform. We performed unsupervised consensus clustering based on the expression levels of 48 genes related to *CTLA-4, PD-1*/*PD-L1*, and cytotoxic lymphocyte (CL). We then investigated the different clinical and immunological characteristics of different immune molecular subtypes (IMMSs) of ovarian cancer. In addition, we explored the relationship between IMMS and TMIT.

## Materials and Methods

### Datasets and Gene Set

Gene expression and clinical data of eight public ovarian cancer datasets (GSE2109, GSE9891, GSE18520, GSE19829, GSE20565, GSE26193, GSE30161, and GSE44104) measured by Affymetrix HG-U133A 2.0 were extracted from the R curatedOvarianData Bioconductor package (19–25). After removal of healthy people, 907 ovarian cancer patients were enrolled in this study. We analyzed 30 CTLA-4-related genes, 9 PD-1/PD-L1-related genes, 6 genes related to both, and 3 CL-related genes. Genes related to anti-CTLA-4 therapy were *CTLA-4, PD-L2, GZMA*, and *PRF1*, as reported by Van Allen et al. (13). Genes related to anti-PD-1/PD-L1 therapy were *PD-L1, CD8A, GZMA*, and *PRF1*, as reported by Ock et al. and Chen et al. (14, 15). TCGA RNA-Seq data were used as independent validation cohort, which included 261 serous ovarian cancer patients.

### Statistical Analysis

The downloaded gene expression data were normalized using the robust multi-array average method (26). Batch effects between different datasets were removed by parametric empirical Bayes method (27). ConsensusClusterPlus R-package was used to conduct unsupervised consensus clustering, which can identify clusters (IMMS) in the expression data (28). The parameters were set as follows: 1,000 iterations, 80% sample resampling, a maximum of 10 clusters, hierarchical clustering with average inner and final linkage, and Pearson correlation as the similarity metric. Kaplan–Meier (K-M) survival curves were used to estimate disease-free survival and overall survival for patients in different IMMSs, and the differences in survival curves were assessed using the log-rank test. The association of clinical characteristics, genes, and disease-free survival were assessed by univariate and multivariate cox regression. Histology type, tumor stage, and grade of different IMMSs were compared using χ^2^ test. One-way analysis of variance was performed to test the difference in gene expression levels of IMMSs. Student–Newman–Keuls-*q* test was used for multiple comparisons. All statistical analyses were performed in the R platform (version 3.3.2). All statistical tests were two-sided and a *P*-value less than 0.05 was considered statistically significant unless specified otherwise.

## Results

In total, 907 ovarian cancer patients from 8 GEO datasets and 48 immunological genes were analyzed in our study (Tables S1 and S2 in Supplementary Material).

### IMMSs Identified by Consensus Clustering

Using the parameters above, consensus clustering can identify 2–10 IMMSs. According to the consensus cumulative distribution function (CDF) plot, cluster-consensus values, and item-consensus values, three IMMSs were identified (Figure S1A in Supplementary Material). These three IMMSs showed the best values, because the CDF reached an approximate maximum (Figures S1B,C in Supplementary Material), and cluster-consensus values (Figure S1D in Supplementary Material) and item-consensus values for one cluster (Figure S1E in Supplementary Material) were large enough to maintain the stable clusters.

### Clinical and Immunological Genes Related to Characteristics of Three IMMSs

Some important clinical characteristics, including age, histology type, stage, and grade, were compared across three IMMSs (Table 1). There was no significant difference in age among the IMMSs; however, differences in histology type, stage, and grade were statistically significant. The proportion of serous ovarian cancer in type I and II ovarian cancer (75.35 and 71.62%, respectively) was similar to the value of 75% reported in previous studies. The proportion of type III ovarian cancer (55.62%) was about 20% lower than 75%, whereas the proportions of other types were higher than those of types I and II to varying extents. Compared with type II and III ovarian cancer, type I cancer had more patients in stage IV and fewer patients in stage I. Regarding tumor grade, there were more patients with grade 3 and fewer patients with grade 1 and 2 in type I ovarian cancer compared with types II and III.

**Table 1:** Clinical characteristics of patients.

Characteristics	IMMS			*F*/χ^2^	*P*-value
			
	I	II	III		
Age	60.66 ± 10.35	59.82 ± 10.94	60.34 ± 11.69	0.24	0.7848
Histology type					
Serous	162 (75.35)	260 (71.63)	183 (55.62)	36.1456	<0.0001
Endometrioid	13 (6.05)	29 (7.99)	32 (9.73)		
Clear cell	5 (2.33)	14 (3.86)	19 (5.78)		
Mucinous	2 (0.93)	8 (2.20)	22 (6.69)		
Other	20 (9.30)	20 (5.51)	34 (10.33)		
Undocumented	13 (6.05)	32 (8.82)	39 (11.85)		
Total	215 (100.00)	363 (100.00)	329 (100.00)		

Stage					
I	11 (5.12)	44 (12.12)	35 (10.64)	15.4011	0.0174
II	20 (9.30)	20 (5.51)	16 (4.86)		
III	126 (58.60)	211 (58.13)	178 (54.10)		
IV	28 (13.02)	29 (7.99)	24 (7.29)		
Undocumented	30 (13.95)	59 (16.25)	76 (23.10)		
Total	215 (100.00)	363 (100.00)	329 (100.00)		

Grade					
1	6 (2.79)	15 (4.13)	15 (4.56)	11.1474	0.0250
2	35 (16.28)	86 (23.69)	75 (22.80)		
3	131 (60.93)	182 (50.14)	140 (42.55)		
Undocumented	43 (20.00)	80 (22.04)	99 (30.09)		
Total	215 (100.00)	363 (100.00)	329 (100.00)		

**Figure 1:**
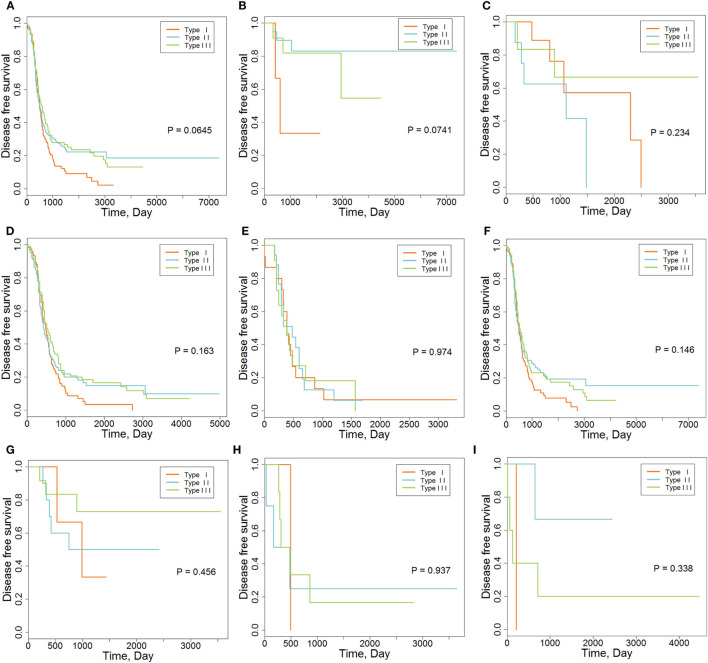
The association between immune molecular subtypes and disease-free survival. **(A)** Kaplan–Meier (K-M) curve for all patients (*N* = 418). **(B)** K-M curve for stage I patients (*N* = 36). **(C)** K-M curve for stage II patients (*N* = 23). **(D)** K-M curve for stage III patients (*N* = 315). **(E)** K-M curve for stage IV patients (*N* = 43). **(F)** K-M curve for serous patients (*N* = 362). **(G)** K-M curve for endometrioid patients (*N* = 26). **(H)** K-M curve for clear cell patients (*N* = 11). **(I)** K-M curve for mucinous patients (*N* = 9).

We also explored the relationships between IMMSs, overall survival, and disease-free survival. As shown in Figure 1A and Figure S2 in Supplementary Material, the differences of survival curves for overall survival and disease-free survival were not statistically significant but patients with type I ovarian cancer tended to have a worse disease-free survival. We further studied the relationship between IMMSs and disease-free survival in different tumor stages and histology types. The differences of survival curves tended to be significant (stage I, stage III, and serous patients) in some subtypes of patients (Figures 1B–I). After adjusting for clinical characteristics and ICB-related genes, IMMSs were associated with disease-free survival (Table 2).

**Table 2:** Univariate and multivariate cox regression analyses of disease-free survival for clinical characteristics and immune checkpoint blockade-related genes.

Variable	Univariate^■^			Multivariate^▾^			Multivariate^•^		
			
	β	*P*-value	HR	β	*P*-value	HR	β	*P*-value	HR
IMMS									
II vs I	−0.256	0.0649	0.774	−0.284	**0.0499**	0.753	–	–	–
III vs I	−0.328	**0.0261**	0.720	−0.392	**0.0235**	0.676	–	–	–
IMMS	−0.288	**0.0223**	0.750	–	–	–	−0.320	**0.0173**	0.726
Age	0.316	**0.0075**	1.371	0.233	0.0585	1.263	0.230	0.0612	1.259
Histology type*									
2 vs 1	−1.148	**0.0004**	0.317	−0.603	0.0664	0.547	−0.603	0.0665	0.547
3 vs 1	0.055	0.8703	1.057	0.570	0.1897	1.769	0.550	0.2043	1.733
4 vs 1	−0.337	0.4159	0.714	0.649	0.1434	1.914	0.668	0.1322	1.951
5 vs 1	−0.154	0.7089	0.857	−0.497	0.2582	0.609	−0.529	0.2253	0.589
6 vs 1	−1.037	0.3007	0.354	−1.183	0.2427	0.306	−1.210	0.2317	0.298
Stage	−1.579	**<0.0001**	0.206	−1.662	**<0.0001**	0.190	−1.653	**<0.0001**	0.191
Grade	0.198	0.0948	1.219	−0.072	0.5586	0.930	−0.067	0.5877	0.935
*CTLA-4*	−0.093	0.4102	0.911	−0.278	0.0462	0.757	−0.290	0.0358	0.748
*PD-L2*	0.028	0.8035	1.029	0.048	0.7153	1.049	0.050	0.7048	1.051
CYT	0.097	0.3920	1.102	−0.173	0.3411	0.841	−0.150	0.4026	0.861
*PD-L1*	0.084	0.4555	1.088	−0.070	0.6396	0.933	−0.057	0.6989	0.944
*CD8A*	0.183	0.1057	1.201	0.293	0.0865	1.341	0.319	0.0553	1.375

*Histology type: 1. Serous, 2. Endometrioid, 3. clear cell, 4. mucinous, 5. other, 6. undocumented. ^■^Univariate cox regression for all variables. ^▾^Multivariate cox regression for all variables when there are three immune molecular subtypes (IMMSs). ^●^Multivariate cox regression for all variables when patients of type II and III ovarian cancer were combined.

*Bold values signifies P value is less than 0.05*.

**Figure 2:**
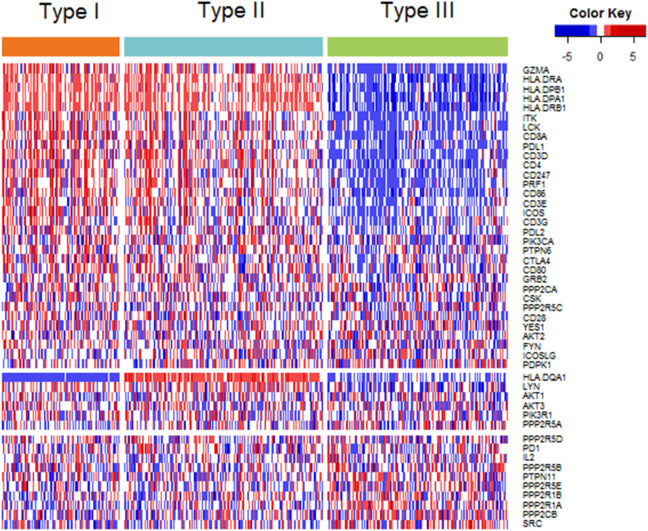
Heatmap plot of 48 genes across 3 immune molecular subtypes (IMMSs). Samples were sorted by IMMSs. Genes were divided into three groups according to highest expression in three IMMSs. Red indicated high expression and blue indicated low expression.

Among the 48 genes in our study, 32 genes showed highest expression in type I ovarian cancer, 6 in type II ovarian cancer, and 10 in type III ovarian cancer (Figure 2). At the level of 0.001, the expression levels of 24, 1, and 3 genes were highest in type I, II, and III, respectively (Table S2 in Supplementary Material). The 24 genes that were highly expressed in type I ovarian were related to *CTLA-4, PD-1*/*PD-L1*, and CL, and the four genes highly expressed in type II and III ovarian cancer were all related to *CTLA-4*.

### Gene Expression Related to ICB in Three IMMSs

**Figure 3:**
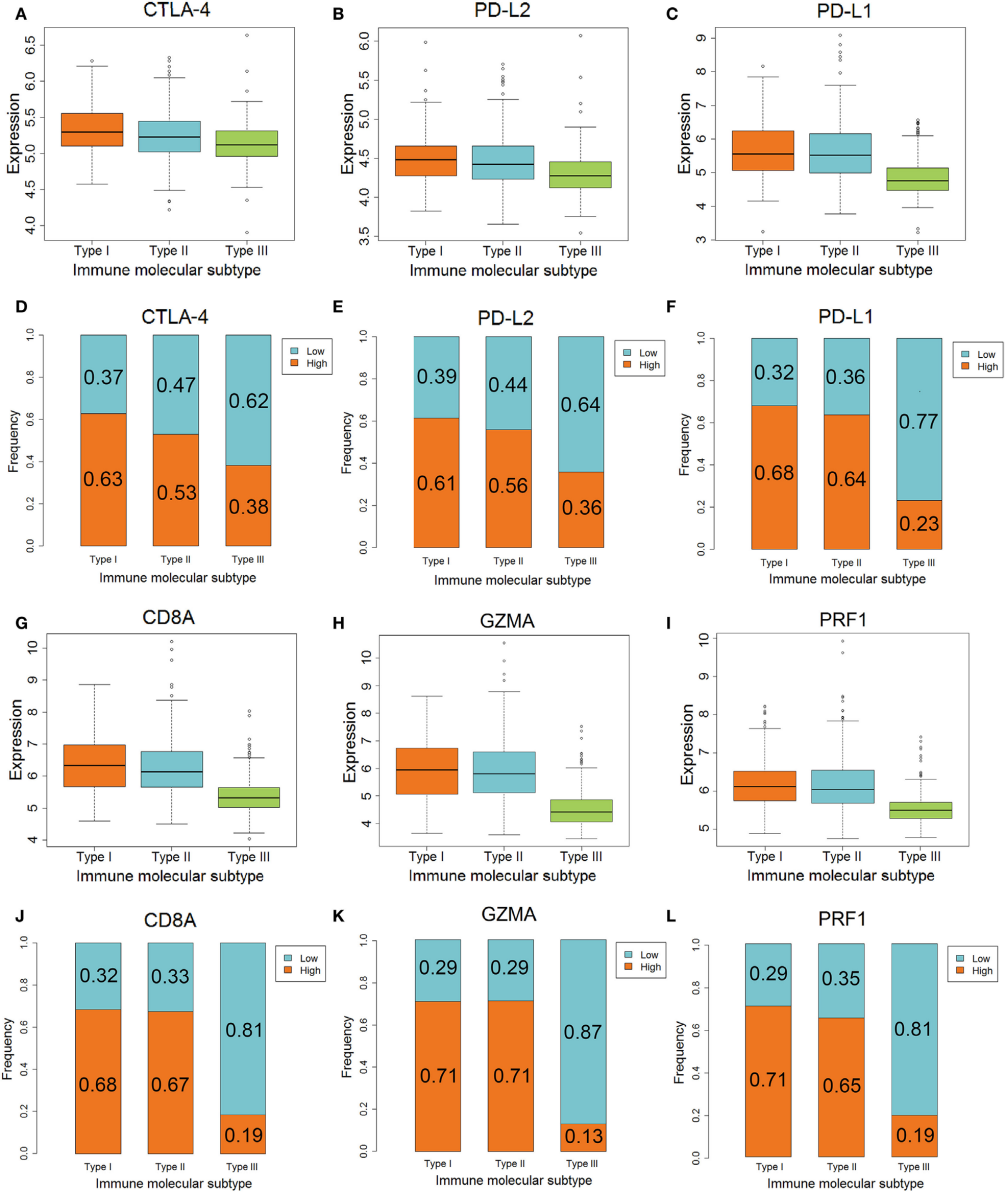
The different expression of genes associated with anti-CTLA-4 and anti-PD-1/PD-L1 therapy among three immune molecular subtypes (IMMSs). **(A)** Boxplot of *CTLA-4* expression across three IMMSs (*P* = 1.97E−12). The expression levels were different in each two IMMSs. **(B)** Boxplot of *PD-L2* across three IMMSs (*P* = 7.67E−15). The expression level in type I and II ovarian cancer was higher than type III ovarian cancer. **(C)** Boxplot of *PD-L1* expression across three IMMSs (*P* = 1.52E−34). The expression level in type I and II ovarian cancer was higher than type III ovarian cancer. **(D)** Percentage bar chart of low- and high expression of *CTLA-4* across 3 IMMSs (*P* = 5.86E−8). **(E)** Percentage bar chart of low- and high expression of *PD-L2* across three IMMSs (*P* = 5.76E−10). **(F)** Percentage bar chart of low- and high expression of *PD-L1* across three IMMSs (*P* < 2.2E−16). **(G)** Boxplot of *CD8A* across three IMMSs (*P* = 3.98E−45). The expression level in type I and II ovarian cancer was higher than type III ovarian cancer. **(H)** Boxplot of *GZMA* across three IMMSs (*P* = 9.43E−61). The expression level in type I and II ovarian cancer was higher than type III ovarian cancer. **(I)** Boxplot of *PRF1* across three IMMSs (*P* = 4.01E−40). The expression level in type I and II ovarian cancer was higher than type III ovarian cancer. **(J)** Percentage bar chart of low- and high expression of *CD8A* across three IMMSs (*P* < 2.2E−16). **(K)** Percentage bar chart of low- and high expression of *GZMA* across three IMMSs (*P* < 2.2E−16). **(L)** Percentage bar chart of low- and high expression of *PRF1* across three IMMSs (*P* < 2.2E−16).

The differences in *CTLA-4, PD-L2, GZMA*, and *PRF1* genes, which are related to anti-CTLA-4 therapy, among the three IMMSs were statistically significant. The expression of *CTLA-4* was highest in type I ovarian cancer followed by type II, and was lowest in type III ovarian cancer (Figure 3A). The expression of *PD-L2, GZMA*, and *PRF1* in type I and II ovarian cancer was higher than that in type III ovarian cancer (Figures 3B,H,I). After dividing the patients into low- and high-expression groups by median expression values, there were more high-expression patients in types I and II compared with type III ovarian cancer (Figures 3D,E,K,L). These results indicate that type I and II ovarian cancer may respond to anti-CTLA-4 therapy better than type III ovarian cancer.

The differences in *PD-L1, CD8A, GZMA*, and *PRF1* genes, which are related to anti-PD-1/PD-L1 therapy, in the three IMMSs were also statistically significant. The expression of each gene in type I and II ovarian cancer was higher than that in type III ovarian cancer (Figures 3C,G,H,I).

As shown in Figures 3F,J,K,L, the proportion of high-expression patients in type I and II ovarian cancer (about 60–70%) was obviously higher than that in type III ovarian cancer (about 10–20%). The higher expression of *PD-L1, CD8A, GZMA*, and *PRF1* in type I and II ovarian cancer indicated that these patients would tend to gain more benefit from anti-PD-1/PD-L1 therapy.

### The Association Between IMMS and TMIT

**Figure 4:**
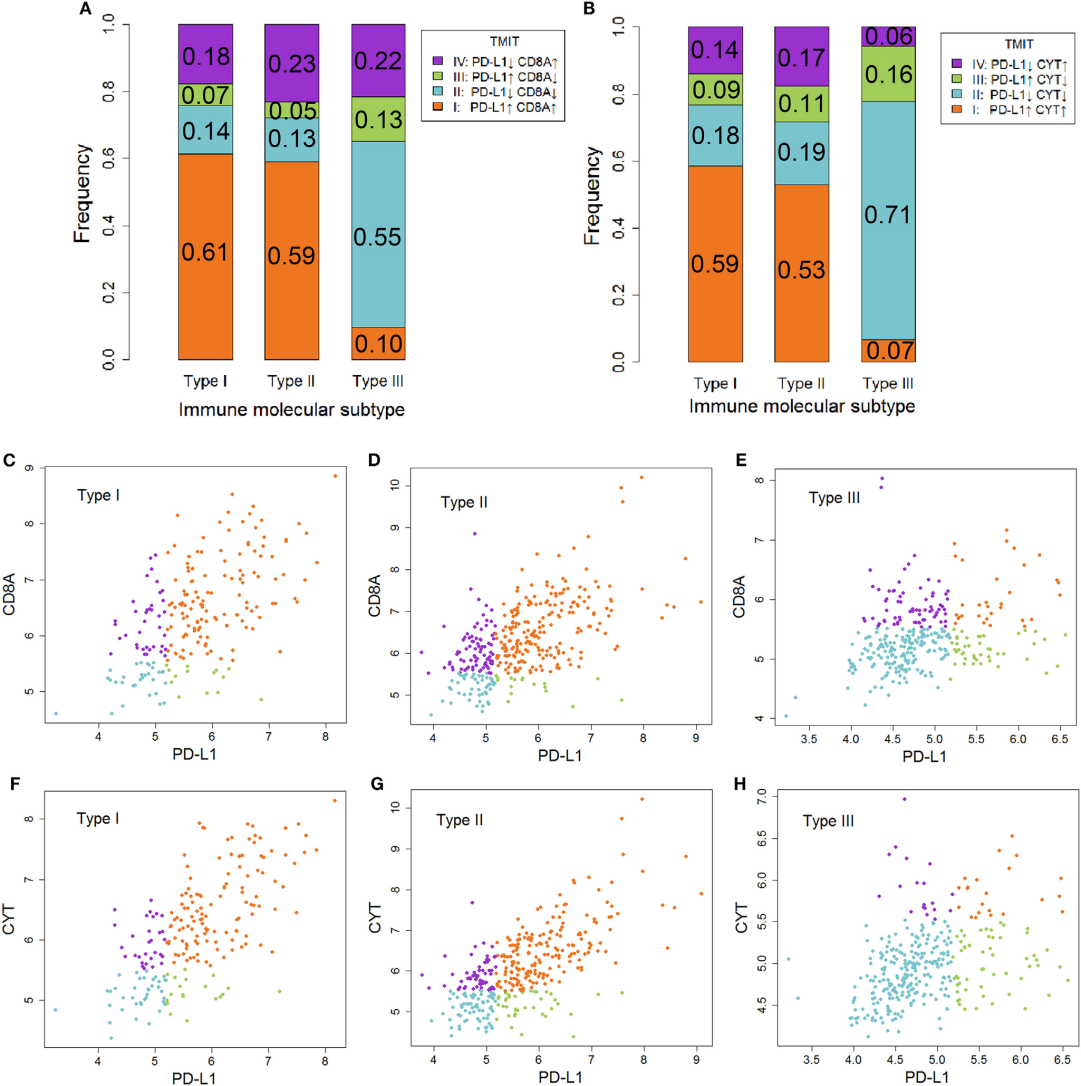
The association between immune molecular subtype (IMMS) and tumor microenvironment immune types (TMIT). **(A)** A summary of four TMITs (*PD-L1* + *CD8A*) in three IMMSs. **(B)** A summary of four TMITs (*PD-L1* + CYT) in three IMMSs. **(C)** Scatter plot of four TMITs (*PD-L1* + *CD8A*) in type I ovarian cancer. **(D)** Scatter plot of four TMITs (*PD-L1* + *CD8A*) in type II ovarian cancer. **(E)** Scatter plot of four TMITs (*PD-L1* + *CD8A*) in type III ovarian cancer. **(F)** Scatter plot of four TMITs (*PD-L1* + CYT) in type I ovarian cancer. **(G)** Scatter plot of four TMITs (*PD-L1* + CYT) in type II ovarian cancer. **(H)** Scatter plot of four TMITs (*PD-L1* + CYT) in type III ovarian cancer.

Tumor microenvironment immune types I cancer was characterized as sensitive to anti-CTLA-4 therapy. In contrast, TMIT II cancer tended to be insensitive to this therapy. As shown in Figure 4, the proportion of TMIT I cancer in type I and II ovarian cancer was about 50–60% whereas the proportion in type III ovarian cancer was less than 10%. For TMIT II cancer, the proportion in type I and II ovarian cancer was about 10–20%, which was much lower than that in type III ovarian cancer (50–70%). These results also supported the notion that type I and II ovarian cancer is more sensitive to anti-CTLA-4 therapy than type III ovarian cancer.

### Validation of IMMS in TCGA RNA-Seq Cohort

We further validated IMMSs by applying the same stratification criteria in TCGA validation cohort. As a result, 126, 19, and 116 patients were identified as type I, II, and III, respectively. Except for the small sample size of type II ovarian cancer, the expression pattern of patients in validation cohort was highly consistent with our findings (Figure S3 in Supplementary Material). Genes related with ICB, including *CTLA-4, PD-L2, PD-L1, CD8A, GZMA*, and *PRF1*, were all lowly expressed in type III ovarian cancer (Figure S4 in Supplementary Material). Besides, fewer TMIT I individuals were observed in type III ovarian cancer (Figure S5 in Supplementary Material).

## Discussion

Immune checkpoint blockade was an important breakthrough for cancer immunotherapy in recent years (2). The application of ICB in ovarian cancer is currently being examined. However, like other tumors, many patients with ovarian cancer will be insensitive to ICB. Therefore, understanding the immunological characteristics of ovarian cancer is critical for ICB and identification of patients who are sensitive to ICB. In our study, we clustered ovarian cancer patients into three IMMSs with different clinical and immunological characteristics. There were more serous and low-grade tumors in type I and II ovarian cancer. Among the differentially expressed genes identified in our study, about 90% (25/28) were highly expressed in type I and II ovarian cancer. All analyses, including immune gene markers studied, cytotoxic function, and TMIT classification, suggested that type I and II categories of patients were similar to each other and distinct from type III. This result suggested that the pathogenesis of type III ovarian cancer may be dominated by other genes and pathways.

Despite the similarity of type I and II ovarian cancer, HLA-DQA1 showed a completely different expression pattern, in which the expression level in type II ovarian cancer was obviously higher than that in type I ovarian cancer. HLA-DQA1 encoded α chain of receptor protein HLA-DQ on APC. Previous studies have proved the expression of HLA-DQA1 decreased in cancer tissue compared with corresponding adjacent tissue and high expression of it was related with better prognosis (29, 30). This phenomenon may explain the shorter disease-free survival time of type I ovarian cancer.

**Figure 5:**
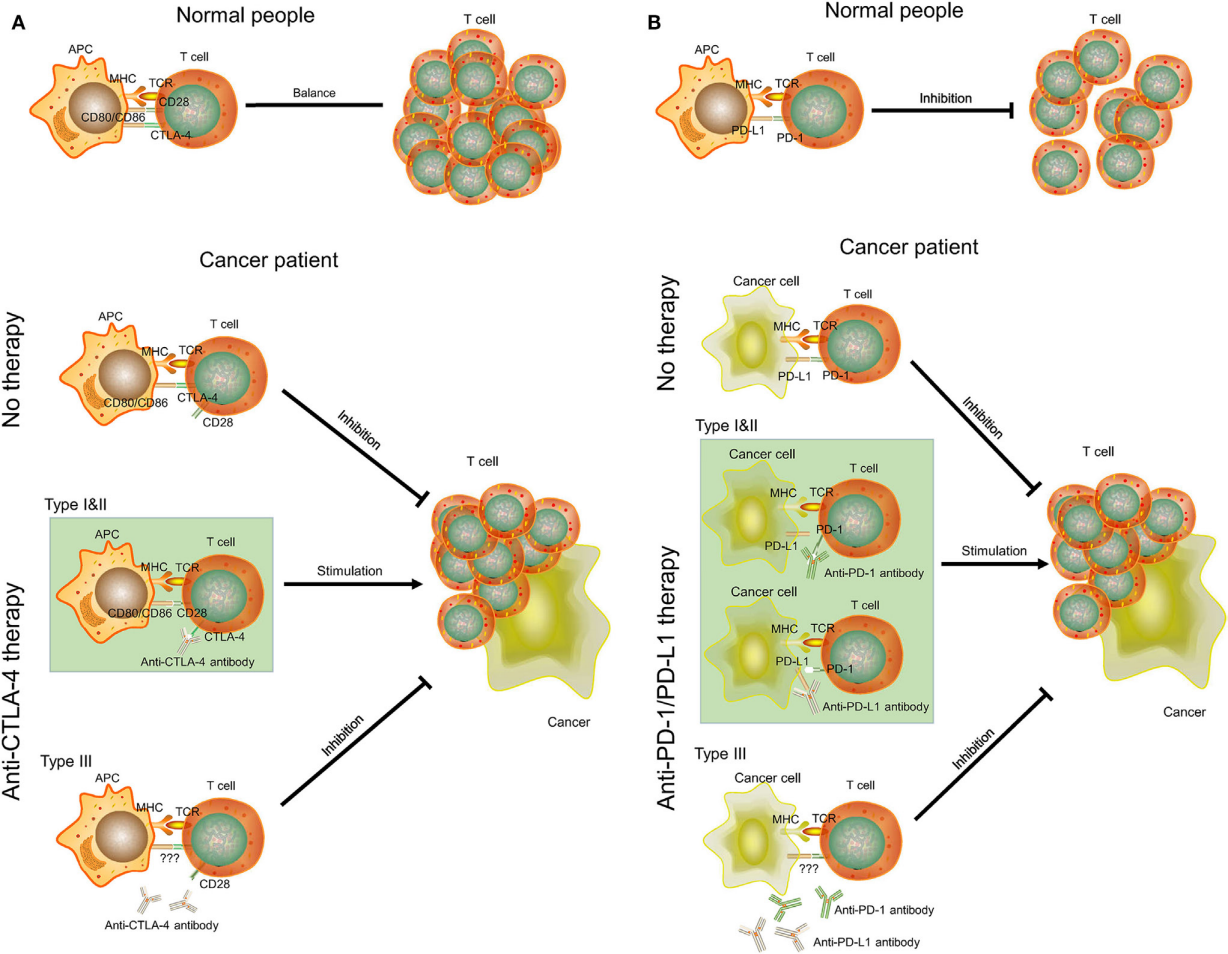
The mechanisms of *CTLA-4, PD-1*/*PD-L1*, anti-CTLA-4, and anti-PD-1/PD-L1 therapy in cancer. **(A)** *CTLA-4* and anti-CTLA-4 therapy. **(B)** *PD-1*/*PD-L1* and anti-PD-1/PD-L1 therapy. APC, antigen-presenting cell; MHC, major histocompatibility complex; TCR, T-cell receptor.

*CTLA-4* is a transmembrane receptor on T cells that shares the B7 ligand (CD80/CD86) with CD28 but binds with a higher avidity (Figure 5A) (31). In contrast to the function of CD80/CD86, *CTLA-4* can transmit an inhibitory signal to T cells to avoid some autoimmune diseases in normal people (32). However, the increase of the expression level of CTLA-4 in some cancer patients (type I and II ovarian cancer) will block the signaling pathway of CD28 and B7 (33). As a result, CD28 cannot promote T-cell activation to inhibit proliferation of cancer cells. Anti-CTLA-4 antibody selectively acts on CTLA-4 in these patients. B7 can bind to CD28 to promote T-cell proliferation and kill cancer cells (34). However, cancer patients with low-expression levels of *CTLA-4* (type III ovarian cancer) will be not sensitive to anti-CTLA-4 therapy. There may also be other mechanisms of anti-CTLA-4 therapy. For example, some antibodies can remove tumor-induced regulatory T cells (35). Yervoy, the first anti-CTLA-4 antibody, can deplete regulatory T cells by engaging *ex vivo* Fc gamma receptor IIIA-expressing, non-classical monocytes in melanoma patients (36).

*PD-1* is a member of the CD28 family on T cells. *PD-L1*, also known as *CD274* and *B7-H1*, is the ligand of *PD-1* and is often highly expressed on antigen-presenting cells (37). The binding of *PD-1* and *PD-L1* plays an important role in downregulating the immune system and promoting self-tolerance of T-cell inflammatory activity (Figure 5B). *PD-L1* was upregulated in ovarian cancers, and anti-PD-L1 therapy could increase the proportion of CD4+ and CD8+ T cell (about 20%) and reduce regulatory T cell (about 5%) in mouse ovarian cancer model to promote tumor rejection (38, 39). Some studies also indicated that combination treatment can further improve antitumor microenvironment, including *PD-1*/*PD-L1* blockade plus *CTLA-4* blockade, vaccine, or irradiation (39–41). However, this effect may be positively related to expression level of *PD-L1*. Cancer cells with high expression of *PD-L1* (type I and II ovarian cancer) may inhibit T-cell proliferation by binding PD-1 on T cells. Thus, anti-PD-L1 antibodies can bind to *PD-L1* on cancer cells and T cells to promote T-cell proliferation. As for type III ovarian cancers with low-expression level of *PD-L1*, anti-PD-1/PD-L1 therapy may not be successful and a combination treatment would be considered.

The expression of genes related with tumor microenvironment (*CD8A, GZMA*, and *PRF1*) can also affect anti-CTLA-4 and anti-PD-1/PD-L1 therapy (17, 42). The expression level of these genes was increased in type I and II ovarian cancer, indicating an appropriate microenvironment for anti-CTLA-4 and anti-PD-1/PD-L1 therapy. However, the tumor microenvironment in type III ovarian cancers may decrease the sensitivity of therapy.

The targets of anti-CTLA-4 and anti-PD-1/PD-L1 therapy described here are all members of the B7-CD28 superfamily (43). Therefore, a synergistic effect might be achieved by combination of these agents. In our study, the expression patterns of genes associated with anti-CTLA-4 therapy, anti-PD-1/PD-L1, and TILs were highly consistent: all genes were highly expressed in type I and II ovarian cancer. Thus, these patients can benefit from combined therapy of anti-CTLA-4 and anti-PD-1/PD-L1 therapy or other immunotherapies.

There were several limitations in our study. The most prominent limitation is that some type I and II ovarian cancer patients may also be insensitive to anti-CTLA-4 and anti-PD-1/PD-L1 therapy. There was no gold standard for these patients who did not undergo ICB, therefore their real responses to the therapy were unknown. We used the expression of ICB-related genes and TMIT to evaluate ICB sensitivity. Our results can only identify those patients who are more likely to be sensitive to ICB.

In conclusion, we used unsupervised consensus clustering to identify three IMMSs of ovarian cancer with different clinical and immunological characteristics. Types I and II with more serous and low-grade tumors were similar to each other and had higher expression levels of genes related to ICB. These patients were likely to be more sensitive to anti-CTLA-4 therapy, anti-PD-1/PD-L1 therapy, and combination immunotherapy. However, the expression of these genes in type III ovarian cancer was low, suggesting that patients with type III disease may be insensitive to these treatments. New therapies for this group of patients should be explored. Overall, our study provides new information for the selection of patients suitable for ICB and personalized therapy of ovarian cancer.

## Author Contributions

KY and WZ wrote the manuscript. KY, GL, and ZR downloaded and analyzed the data. WZ, HX, and WW prepared all the figures and tables. KL and YH designed the entire study and edited the manuscript. All authors reviewed and approved the final manuscript.

## Conflict of Interest Statement

The authors declare that the research was conducted in the absence of any commercial or financial relationships that could be construed as a potential conflict of interest.
