# Supplementary material for: An Immunophenotyping of Ovarian Cancer With Clinical and Immunological Significance
Source: Front Immunol. 2018 Apr 10;9:757. doi: 10.3389/fimmu.2018.00757 (PMC7394551; doi:10.3389/fimmu.2018.00757)
Supplement: Supplementary file 1 [file Table_1.DOCX]

**Table S1.** Number of patients of different datasets in this study.

| GEO ID | Number of patients |
| --- | --- |
| GSE2109 | 194 |
| GSE9891 | 267 |
| GSE18520 | 53 |
| GSE19829 | 28 |
| GSE20565 | 140 |
| GSE26193 | 107 |
| GSE30161 | 58 |
| GSE44104 | 60 |
| Total | 907 |
