# Supplementary material for: An Immunophenotyping of Ovarian Cancer With Clinical and Immunological Significance
Source: Front Immunol. 2018 Apr 10;9:757. doi: 10.3389/fimmu.2018.00757 (PMC7394551; doi:10.3389/fimmu.2018.00757)
Supplement: Supplementary file 2 [file Table_2.DOCX]

**Table S2.** Results of One-way analysis of variance for 48 genes.

| Gene | Related term | *P* | Gene | Related term | *P* |
| --- | --- | --- | --- | --- | --- |
| AKT1 | CTLA-4 | 8.16E-03 | PPP2R5C | CTLA-4 | 9.78E-03 |
| AKT2 | CTLA-4 | 6.03E-01 | PPP2R5D | CTLA-4 | 5.32E-01 |
| AKT3 | CTLA-4 | 2.30E-01 | PPP2R5E | CTLA-4 | 1.40E-02 |
| CD28 | CTLA-4 | 2.26E-02 | PTPN11 | CTLA-4 | 5.29E-03 |
| CD80 | CTLA-4 | 3.09E-09 | SRC | CTLA-4 | 6.97E-10 |
| CD86 | CTLA-4 | 3.05E-53 | YES1 | CTLA-4 | 1.80E-01 |
| CTLA-4 | CTLA-4 | 1.97E-12 | PD-L1 | PD-1 | 1.52E-34 |
| FYN | CTLA-4 | 3.50E-01 | CD4 | PD-1 | 2.63E-59 |
| GRB2 | CTLA-4 | 1.83E-05 | CSK | PD-1 | 6.65E-03 |
| HLA-DRA | CTLA-4 | 2.18E-119 | HLA-DPA1 | PD-1 | 2.40E-121 |
| ICOS | CTLA-4 | 1.13E-24 | HLA-DPB1 | PD-1 | 1.22E-115 |
| ICOSLG | CTLA-4 | 9.37E-01 | HLA-DQA1 | PD-1 | 9.01E-03 |
| IL2 | CTLA-4 | 7.00E-01 | PD-1 | PD-1 | 6.04E-01 |
| ITK | CTLA-4 | 1.71E-43 | PD-L2 | PD-1 | 7.67E-15 |
| LYN | CTLA-4 | 3.36E-27 | PTPN6 | PD-1 | 1.97E-11 |
| PDPK1 | CTLA-4 | 9.63E-01 | CD247 | Both | 6.61E-43 |
| PIK3CA | CTLA-4 | 1.04E-07 | CD3D | Both | 6.84E-55 |
| PIK3R1 | CTLA-4 | 3.24E-01 | CD3E | Both | 1.15E-31 |
| PPP2CA | CTLA-4 | 3.63E-04 | CD3G | Both | 4.34E-21 |
| PPP2CB | CTLA-4 | 8.48E-05 | HLA-DRB1 | Both | 9.40E-122 |
| PPP2R1A | CTLA-4 | 1.57E-04 | LCK | Both | 6.62E-35 |
| PPP2R1B | CTLA-4 | 4.89E-02 | GZMA | CL | 9.43E-61 |
| PPP2R5A | CTLA-4 | 2.80E-01 | PRF1 | CL | 4.01E-40 |
| PPP2R5B | CTLA-4 | 3.01E-01 | CD8A | CL | 3.98E-45 |
