# Supplementary material for: An Immunophenotyping of Ovarian Cancer With Clinical and Immunological Significance
Source: Front Immunol. 2018 Apr 10;9:757. doi: 10.3389/fimmu.2018.00757 (PMC7394551; doi:10.3389/fimmu.2018.00757)
Supplement: Supplementary file 3 [file Image_1.PDF]

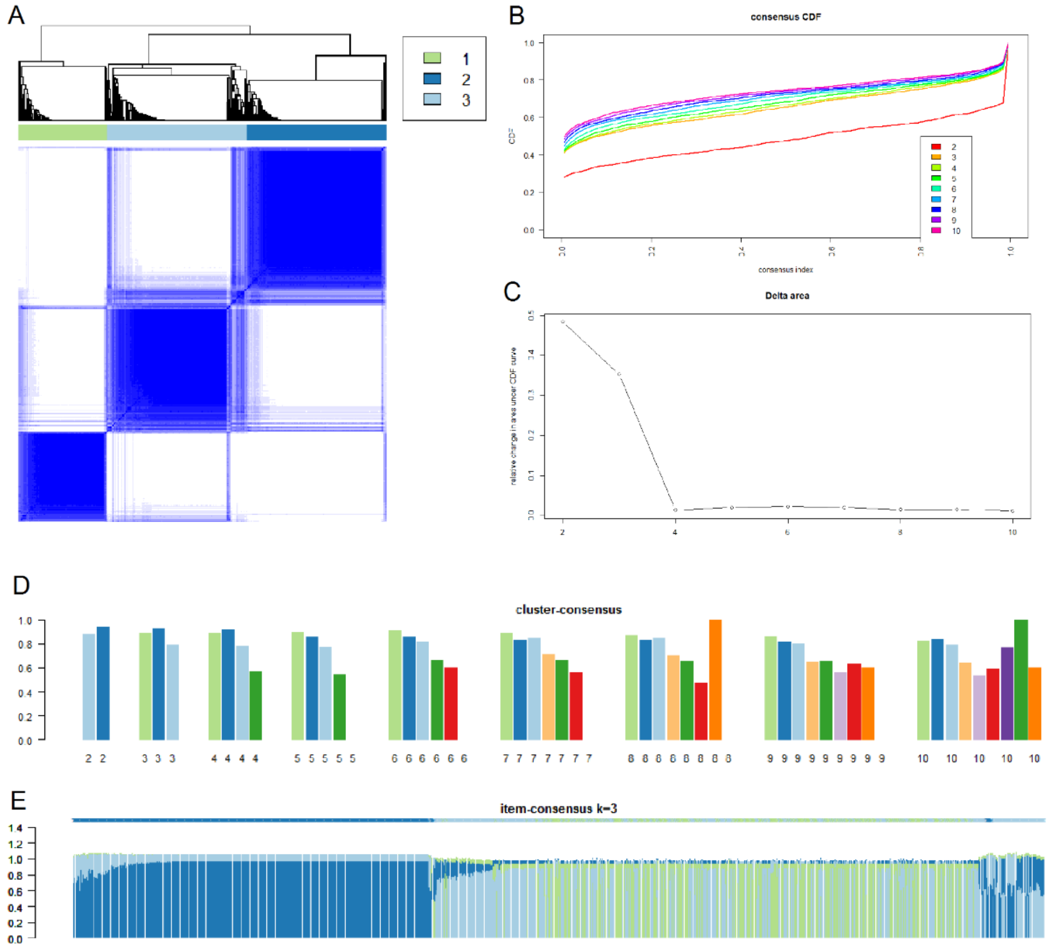

Figure S1. The results of unsupervised consensus clustering. **(A)** Heatmap of the consensus matrices for 3 clusters. **(B)** The cumulative distribution functions (CDF) of the consensus matrix for 2 to 10 clusters (indicated by colors). **(C)** The relative change in area under the CDF curve comparing clusters and clusters-1. **(D)** The cluster-consensus value of each clusters at 2 to 10 clusters. **(E)** Item-consensus values at 3 clusters.
