# Supplementary figures and images for: An Immunophenotyping of Ovarian Cancer With Clinical and Immunological Significance
Source: Front Immunol. 2018 Apr 10;9:757. doi: 10.3389/fimmu.2018.00757 (PMC7394551; doi:10.3389/fimmu.2018.00757)

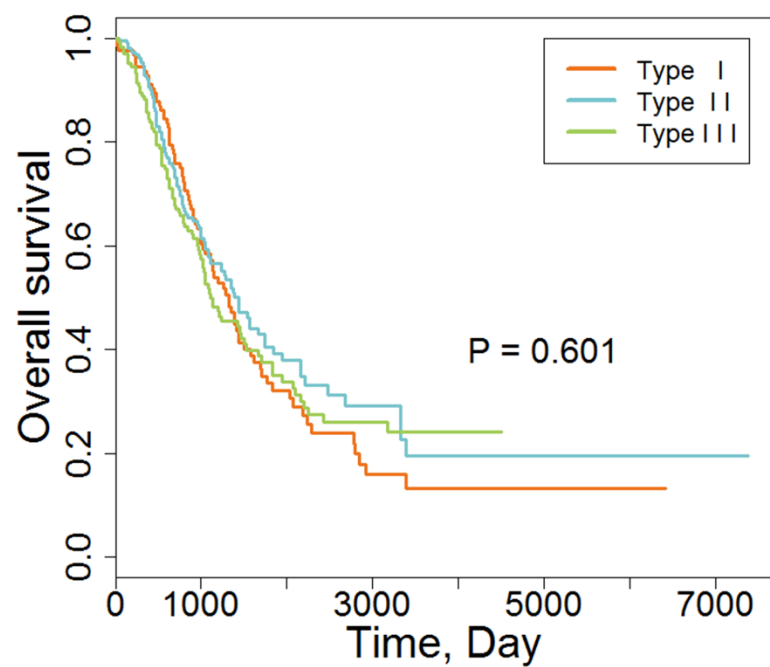

**Figure S2.** K-M curve of overall survival among 3 IMMSs.

Supplement: Supplementary file 4 [file Image_2.PDF]
