# Supplementary material for: An Immunophenotyping of Ovarian Cancer With Clinical and Immunological Significance
Source: Front Immunol. 2018 Apr 10;9:757. doi: 10.3389/fimmu.2018.00757 (PMC7394551; doi:10.3389/fimmu.2018.00757)
Supplement: Supplementary file 5 [file Image_3.PDF]

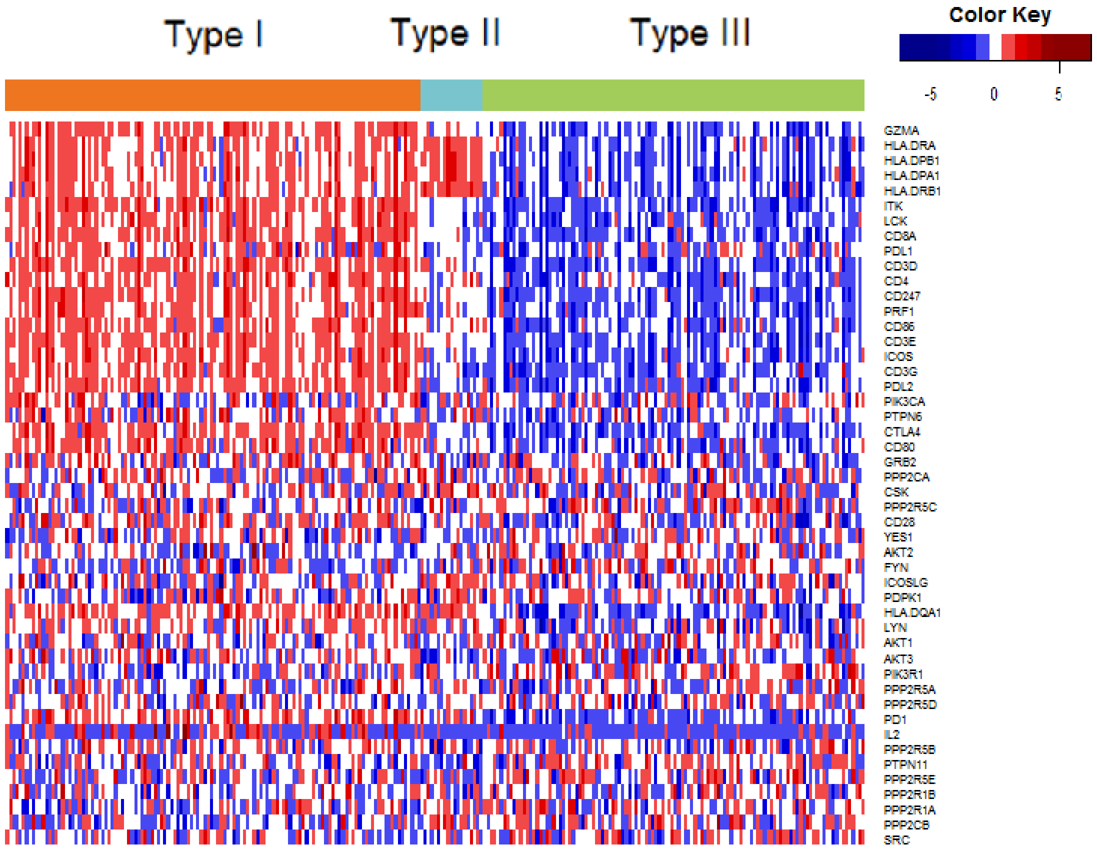

Figure S3. Heatmap plot of 48 genes across 3 IMMSs in the TCGA validation cohort. Red indicated high expression and blue indicated low expression.
