# Supplementary material for: An Immunophenotyping of Ovarian Cancer With Clinical and Immunological Significance
Source: Front Immunol. 2018 Apr 10;9:757. doi: 10.3389/fimmu.2018.00757 (PMC7394551; doi:10.3389/fimmu.2018.00757)
Supplement: Supplementary file 6 [file Image_4.PDF]

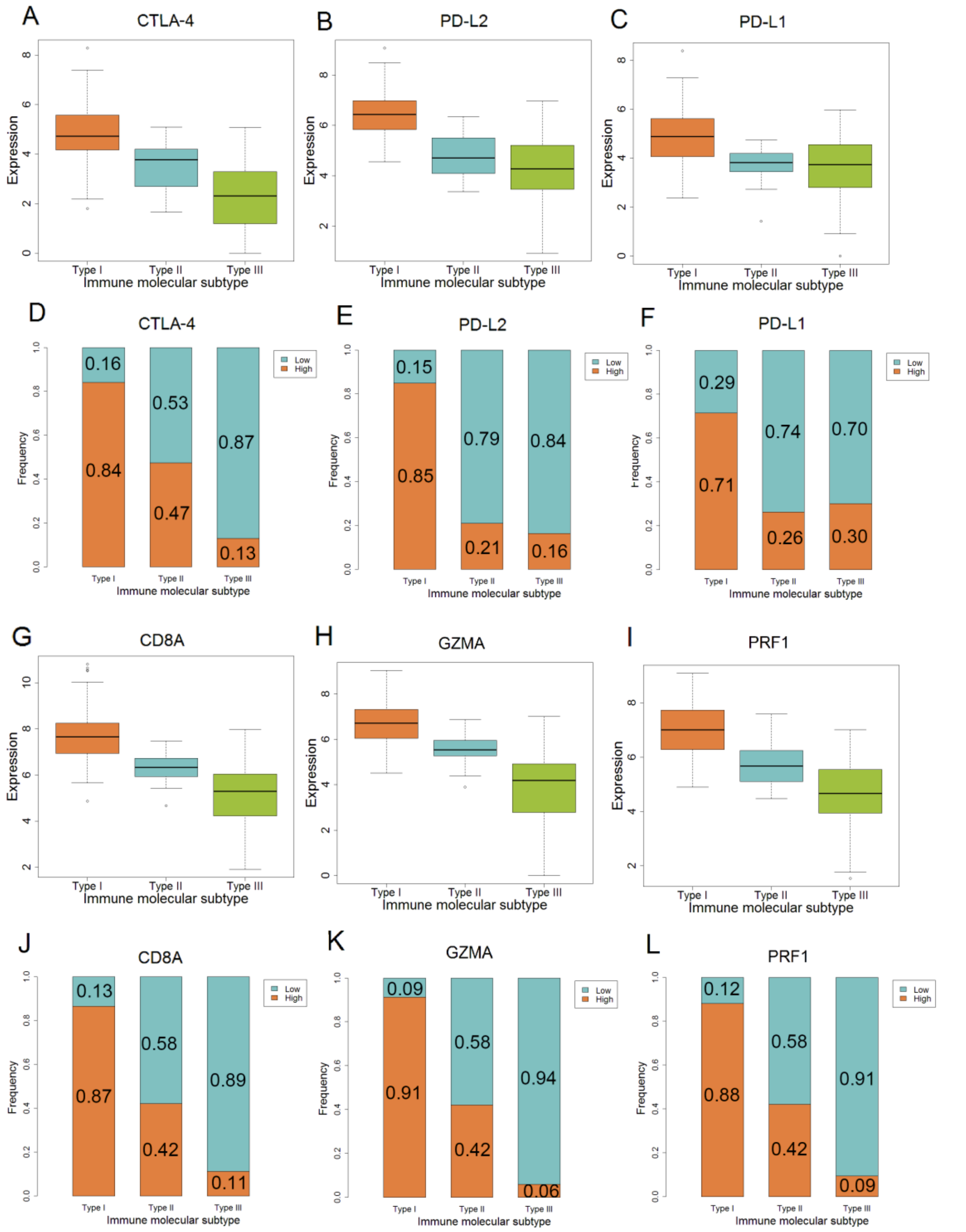

Figure S4. The different expression of genes associated with anti-CTLA-4 and anti-PD-1/PD-L1 therapy among 3 IMMSs in the TCGA validation cohort. **(A)** Boxplot of *CTLA-4* expression across 3 IMMSs (P = 1.19E-43). The expression levels were different in each two IMMSs. **(B)** Boxplot of *PD-L2* across 3 IMMSs (P = 2.60E-39). The expression levels were different in each two IMMSs. **(C)** Boxplot of *PD-L1* expression across 3 IMMSs (P = 1.59E-14). The expression level in type I ovarian cancer was higher than type II&III ovarian cancer. **(D)** Percentage bar chart of low- and high-expression of *CTLA-4* across 3 IMMSs (P < 2.2E-16). **(E)** Percentage bar chart of

low- and high-expression of *PD-L2* across 3 IMMSs (*P* < 2.2E-16). **(F)** Percentage bar chart of low- and high-expression of *PD-L1* across 3 IMMSs (*P* = 1.2E-10). **(G)** Boxplot of *CD8A* across 3 IMMSs (*P* = 8.33E-47). The expression levels were different in each two IMMSs. **(H)** Boxplot of *GZMA* across 3 IMMSs (*P* = 2.47E-49). The expression levels were different in each two IMMSs. **(I)** Boxplot of *PRF1* across 3 IMMSs (*P* = 5.56E-48). The expression levels were different in each two IMMSs. **(J)** Percentage bar chart of low- and high-expression of *CD8A* across 3 IMMSs (*P* < 2.2E-16). **(K)** Percentage bar chart of low- and high-expression of *GZMA* across 3 IMMSs (*P* < 2.2E-16). **(L)** Percentage bar chart of low- and high-expression of *PRF1* across 3 IMMSs (*P* < 2.2E-16).
