# Supplementary material for: An Immunophenotyping of Ovarian Cancer With Clinical and Immunological Significance
Source: Front Immunol. 2018 Apr 10;9:757. doi: 10.3389/fimmu.2018.00757 (PMC7394551; doi:10.3389/fimmu.2018.00757)
Supplement: Supplementary file 7 [file Image_5.PDF]

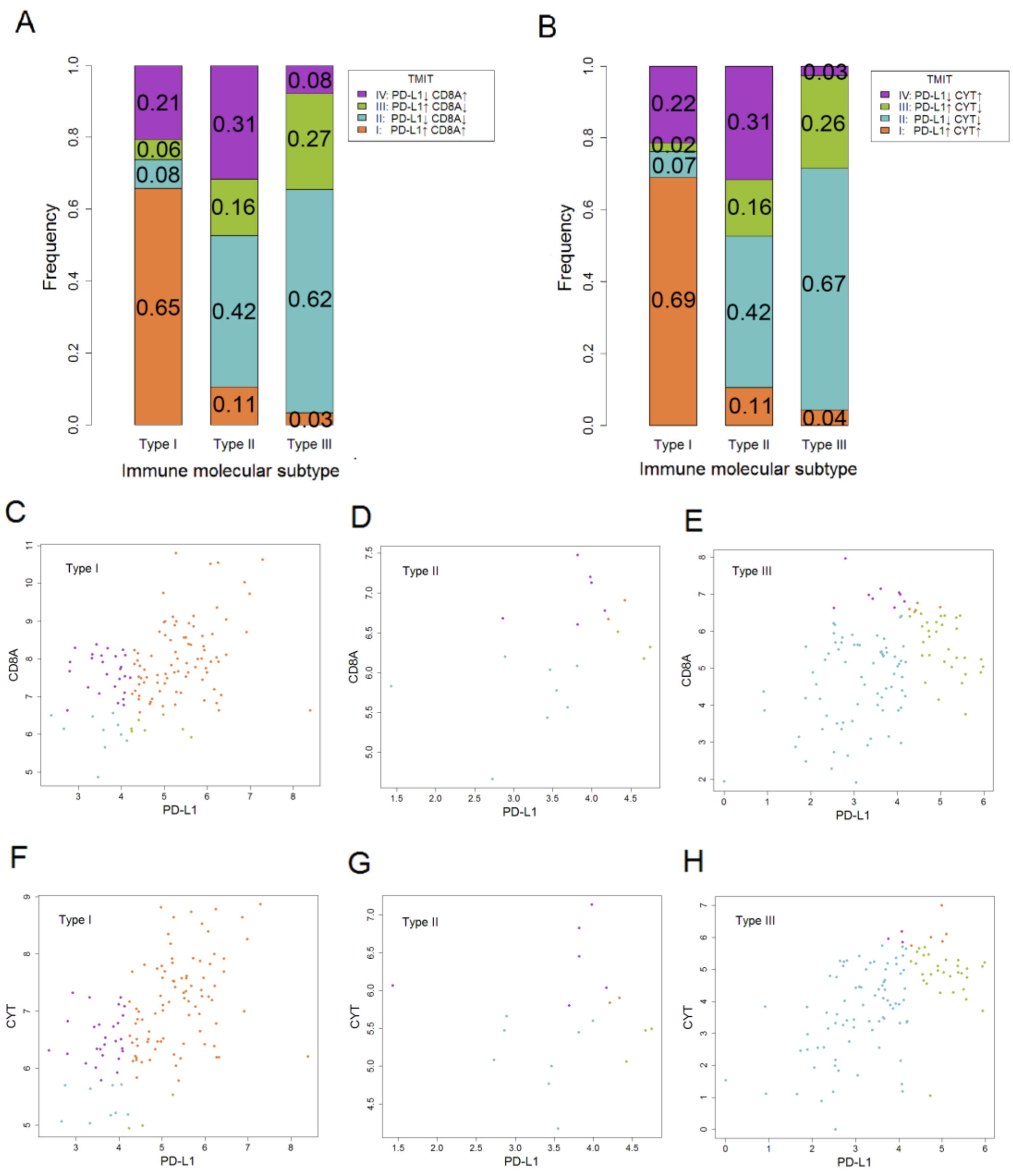

Figure S5. The association between IMMS and TMIT in the TCGA validation cohort. **(A)** A summary of 4 TMITs (*PD-L1* + *CD8A*) in 3 IMMSs. **(B)** A summary of 4 TMITs (*PD-L1* + CYT) in 3 IMMSs. **(C)** Scatter plot of 4 TMITs (*PD-L1* + *CD8A*) in type I ovarian cancer. **(D)** Scatter plot of 4 TMITs (*PD-L1* + *CD8A*) in type II ovarian cancer. **(E)** Scatter plot of 4 TMITs (*PD-L1* + *CD8A*) in type III ovarian cancer. **(F)** Scatter plot of 4 TMITs (*PD-L1* + CYT) in type I ovarian cancer. **(G)** Scatter plot of 4 TMITs (*PD-L1* + CYT) in type II ovarian cancer. **(H)** Scatter plot of 4 TMITs (*PD-L1* + CYT) in type III ovarian cancer.
